# Dinosaur Fossils Predict Body Temperatures

**DOI:** 10.1371/journal.pbio.0040248

**Published:** 2006-07-11

**Authors:** James F Gillooly, Andrew P Allen, Eric L Charnov

**Affiliations:** **1**Department of Zoology, University of Florida, Gainesville, Florida, United States of America; **2**National Center for Ecological Analysis and Synthesis, Santa Barbara, California, United States of America; **3**Department of Biology, University of New Mexico, Albuquerque, New Mexico, United States of America; **4**Department of Fisheries and Wildlife, Oregon State University, Corvallis, Oregon, United States of America; University of California BerkeleyUnited States of America

## Abstract

Perhaps the greatest mystery surrounding dinosaurs concerns whether they were endotherms, ectotherms, or some unique intermediate form. Here we present a model that yields estimates of dinosaur body temperature based on ontogenetic growth trajectories obtained from fossil bones. The model predicts that dinosaur body temperatures increased with body mass from approximately 25 °C at 12 kg to approximately 41 °C at 13,000 kg. The model also successfully predicts observed increases in body temperature with body mass for extant crocodiles. These results provide direct evidence that dinosaurs were reptiles that exhibited inertial homeothermy.

## Abstract 2

A model based on growth trajectories estimated from fossils provides evidence that dinosaurs were reptiles whose body temperatures increased systematically with increasing body size.

## Introduction

Body temperature regulation in dinosaurs has long been a topic of interest and debate in biology because of its importance to understanding the physiology and life history of these ancient, exceptionally large animals [ 1]. Some have argued that dinosaurs were endotherms with body temperatures that were high, relatively constant, and internally regulated, just as in contemporary birds and mammals (e.g., [ 2]). Others have argued that dinosaurs were reptile-like in their metabolism, but that large dinosaurs maintained higher, more constant body temperatures than smaller-sized reptiles due to thermal inertia (e.g., [ 3, 4]). According to the latter “inertial homeothermy hypothesis,” dinosaur body temperatures were primarily determined by the interaction between environmental temperature and the production and dissipation of heat. The inertial homeothermy hypothesis has thus far been supported by physiological or morphological data from extant ectotherms and endotherms, and by predictions from biophysical models [ 5, 6]. Resolution of the debate regarding body temperature regulation in dinosaurs has thus far been hampered by a lack of direct evidence [ 7].

Here we directly test the inertial homeothermy hypothesis by assessing whether dinosaur body temperatures increased with body size. To estimate body temperatures, we use data on the ontogenetic growth trajectories of eight dinosaur species— *Syntarsus rhodesiensis, Psittacosaurus mongoliensis, Apatosaurus excelsus, Tyrannosaurus rex, Daspletosaurus torosus, Gorgosaurus libratus, Albertosaurus sarcophagus,* and Massospondylus carinatus—that ranged in adult size from 15–25,952 kg, and that lived during the early Jurassic to late Cretaceous periods. These eight growth trajectories were obtained from published work that use newer methods of bone histology and body size estimation [ 8– 11] to estimate the maximum growth rate, *G* (kg day ^−1^), and the mass at maximum growth, *M* (kg), which is about half of the asymptotic adult size (see Materials and Methods).

While data were also available for the dinosaur bird *Shuvuuia deserti,* this species was excluded from our analysis because it is a feathered species and is therefore fundamentally different than the eight more reptile-like species mentioned above.

The recent availability of these data, along with recent advances in understanding the effects of body size and temperature on growth [ 12, 13], allow us to apply a novel approach to estimate dinosaur body temperatures. Specifically, we analyze these data using a recently published model that predicts the combined effects of body size and temperature, *T _b_* (°C), on maximum growth rate [ 12, 13]:



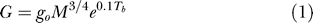



Equation 1 builds on a previously published model that predicts growth rates for a broad assortment of ectotherms and endotherms [ 14]. It has now been used successfully to predict rates of embryonic growth in diverse taxa [ 13], rates of post-embryonic growth in zooplankton [ 13], rates of individual-level biomass production [ 15], and rates of population-level growth in diverse taxonomic groups [ 16]. Here *g _o_* is a normalization constant that is independent of temperature and body size [ 11, 12]. The temperature term, 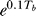, describes the exponential effects of body temperature on whole-organism growth rates. Specifically, it assumes that the biochemical reactions controlling growth have an activation energy of 0.6–0.7 eV, reflecting the temperature dependence of individual metabolic rate [ 17, 18]. The value 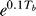 represents the mid-point of this range of activation energies. The use of this temperature term is supported by recent work for a broad assortment of organisms [ 11], and by work conducted near the beginning of the last century (i.e., Krogh's curve) [ 19]. The body size term, *M* ^3/4^, is theoretically predicted [ 14, 20] and empirically supported by extensive data [ 12], including maximum growth-rate data for extant reptiles [ 21] and mammals [ 22]. Given that the coefficient *g _o_* is similar for taxa with different modes of body temperature regulation (∼2 × 10 ^−4^ kg ^1/4^ day ^−1^ for ectotherms and endotherms; see Materials and Methods), we can rearrange the terms in Equation 1 to estimate the body temperature of each dinosaur species as:



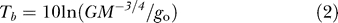



based on its estimated maximum growth rate, *G,* and mass at the time of maximum growth, *M* (see Materials and Methods).

## Results/Discussion

**Figure 1:**
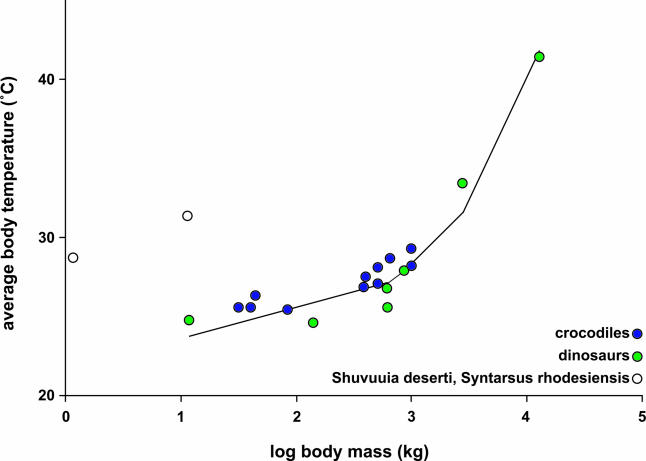
A Plot of the Relationship between Average Body Temperature (°C) and the Logarithm of Body Mass for Dinosaurs and Extant Crocodiles For dinosaurs, body temperatures were estimated from Equation 2 using data on ontogenetic growth trajectories determined from bone histology (see Materials and Methods). Body mass is expressed as the size at which maximum growth rates occur, which is about half of asymptotic adult size. The fitted line includes the following species in ascending order of weight: P. mongoliensis (12 kg), M. carinatus (140 kg), *Al. sarcophagus* (614 kg), G. libratus (622 kg) *, D. torosus* (869 kg), T. rex (2,780 kg), and *Ap. excelsus* (12,979 kg) [ 8– 11]. The line was fit to the data using non-linear least squares regression in order to generate predictions on the change in body temperature with body mass for crocodiles ( Figure 2). This line does not include the two additional species shown here, the dinosaur bird *Sh. deserti* (1 kg), which was not considered because it was feathered, and *Sy. rhodesiensis* (11 kg), which was excluded because it was an outlier (see text). For crocodiles, body temperature estimates are based on the previously observed relative increase in body temperature with body size for individuals (32–1,010 kg) held under natural conditions, and by assuming a mean annual environmental temperature of 25 °C [ 5].

Equation 2 yields body temperature estimates for each of the eight dinosaur species. Results for seven of the eight species indicate that body temperature increases curvilinearly with the logarithm of body size ( Figure 1). The eighth species, *Sy. rhodesiensis,* is clearly an outlier, and is therefore excluded from subsequent analyses (but see discussion below). For the remaining species, body temperature increases by only 2 °C with size from the 12-kg P. mongoliensis to the 614-kg *Al. sarcophagus,* but then increases by nearly 15 °C from the 218-kg *Al. sarcophagus* to the 12, 979-kg *Ap. excelsus*. These results suggest that the smallest dinosaurs, with body temperatures of about 25 °C, had temperatures close to environmental temperatures, as observed for smaller-sized extant reptiles [ 5]. We can characterize this increase in body temperature with size by fitting a non-linear least squares regression model to the data depicted in Figure 1 ( *T _b_* = 23.3 + ( *M*/44.4) ^0.5^, where 23.3 °C, 44.4 kg/°C ^2^, and 0.5 are all fitted parameter estimates). Interestingly, the intercept of this equation, 23.3 °C, is the estimated average environmental temperature for these dinosaurs. This estimate is in agreement with most paleotemperature estimates during the dinosaur age, which generally range between 20 and 30 °C across latitudes [ 5]. We note that our body temperature estimates for dinosaurs should be relatively insensitive to the modest variation that exists in *g _o_* between reptiles and mammals (see Materials and Methods), because the effect of *g _o_* on *T _b_* is only logarithmic in Equation 2. More importantly, the relative increase in body temperature with body mass predicted by the model is entirely independent of *g _o._*

**Figure 2:**
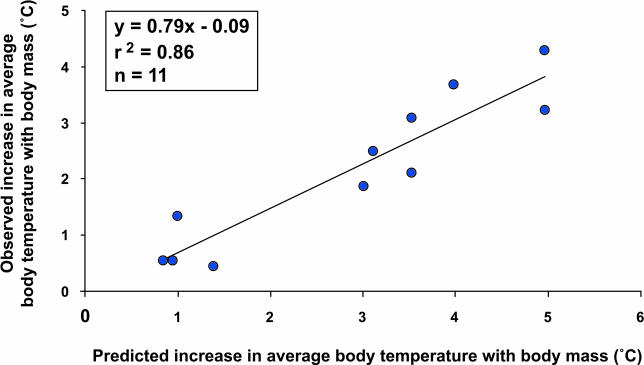
A Plot of the Observed versus the Predicted Increase in Average Body Temperature (°C) with Body Mass for Crocodiles The observed increase in average body temperature for crocodiles ranging in mass from 32–1,010 kg [ 5] was plotted versus the predicted increase in average body temperature with body mass for these crocodiles based on the line fit to the dinosaur data shown in Figure 1 and predicted from Equation 2.

The relationship depicted in Figure 1 also suggests that dinosaur body temperatures changed over the ontogeny of an individual, sometimes dramatically. More specifically, it suggests that body temperatures increased by less than 3 °C over ontogeny for species reaching adult sizes of 300 kg, but by more than 20 °C for species reaching sizes of approximately 25,000 kg, such as *Ap. excelsus*. If we extrapolate the model depicted in Figure 1 up to what is perhaps the largest dinosaur species (∼55,000 kg for an adult Sauroposeidon proteles [ 23]), the estimated body temperature at the mass of maximum growth is approximately 48 °C, which is just beyond the upper limit tolerated for most animals (∼45 °C). These findings suggest that maximum dinosaur size may have ultimately been limited by body temperature.

Model predictions regarding the change in body temperature with body size are strongly supported by a test using data from extant crocodiles ranging in size from 32–1,010 kg. The observed increase in body temperature with size for crocodiles [ 5] appears continuous with our estimates for dinosaurs if we assume that the average annual environmental temperature for crocodiles was 25 °C, close to empirical measures [ 5] ( Figure 1). However, even without making any assumptions about environmental temperature, the relative change in body temperature predicted by the dinosaur model is similar to the empirically observed increase in temperature with size for extant crocodiles (see Materials and Methods). Specifically, a plot of the predicted versus observed change in body temperature with size for crocodiles yields a slope of 0.79, and an *r ^2^* value of 0.86 ( Figure 2). Furthermore, the intercept of this relationship is near 0 (−0.09), suggesting that environmental temperatures for dinosaurs were similar to those of extant crocodiles. Note that using a fitted non-linear regression model that includes the outlier S. rhodesiensis in Figure 1 ( *T _b_* = 26.31 + ( *M*/393.76) ^0.78^) still gives a highly significant relationship between predicted and observed body temperatures for crocodiles ( *r ^2^* = 0.86, *p* < 0.01).

The results presented here provide what is perhaps the first direct evidence that dinosaurs were reptiles whose body temperatures increased systematically with body size, consistent with the inertial homeothermy hypothesis [ 4, 6, 5, 24]. The increase in body temperature with body size shown here for dinosaurs (26–41 °C in Figure 1) is far greater than for any animals living today. This would explain the observation that large dinosaurs grew at rates similar to those of extant eutherian mammals [ 8, 9], which generally maintain body temperatures of 36–38 °C [ 25], but that small dinosaurs grew at rates similar to extant reptiles [ 8, 9], which generally have lower body temperatures of 25–35 °C [ 26] ( Figure 2). In other words, our model and these results indicate that the reason the body size scaling of maximum growth rate may be steeper than *M* ^3/4^ for dinosaurs, but not for reptiles, birds, or mammals [ 8, 9, 27, 28], is largely due to the confounding effects of increasing temperature with increasing body size over this large size range. An increase in body temperature of more than 15 °C from the smallest to largest dinosaurs ( Figure 1) would likely have had important consequences for many aspects of dinosaur life history.

## Materials and Methods

### Estimating size and maximum growth.

Ontogenetic growth curves of dinosaur species were fitted using the equation *m*( *t*) = ( *a/*(1 + exp[ *b*( *t* − *c*)])) + *m _0_* , where *m*( *t*) is the mass at time *t, m _0_* is the mass at *t* = 0, and *a* + *m _0_* is the asymptotic adult mass [ 8, 9]. The fitted parameters in references [ 8, 9] yielded estimates for the maximum growth rates, *G* = − *ab/*4, and the mass at the time of maximum growth, *M* = *a/*2 + *m _0_*. Given the difficulty in estimating *G* and *M,* and possible errors associated with different methodologies, we only included species from references [ 8, 9] where *G* and *M* were estimated using the same methodology. These criteria exclude the one other known species of dinosaur for which a growth trajectory has been established [ 29]. See references [ 8– 11] for more information on the methods used to estimate sizes and ages of individuals.

### Estimating *g _o_*.

The value of *g _o_* used here was estimated from data on the scaling of maximum growth rates for reptiles [ 21] and mammals [ 22]. Linear regression models of the form log( *G*) versus log( *M*) were fitted to both sets of data. The slopes of both relationships include the value predicted by Equation 1 of 0.75 (95% confidence intervals: 0.58–0.84, *n* = 12, for reptiles and 0.69–0.75, *n* = 163, for mammals). Therefore, *g _o_* was separately calculated as the geometric mean of the 12 estimates of *GM* ^−3/4^e ^−0.1 *Tb*^ for reptiles and the 163 estimates of *GM* ^−3/4^e ^−0.1 *Tb*^ for mammals. Taking *T _b_* to be 37 °C for mammals [ 25] and 30 °C for reptiles [ 26] yielded geometric mean estimates for *g _o_* that were remarkably similar for reptiles (1.7 × 10 ^−4^ kg ^1/4^ day ^−1^) and mammals (2.3 × 10 ^−4^ kg ^1/4^ day ^−1^). We therefore used the average of these two estimates (2 × 10 ^−4^ kg ^1/4^ day ^−1^) for our calculations of dinosaur body temperatures.
